# Facilitators and barriers to COVID-19 vaccination among incarcerated people and staff in three large, state prisons: a cross-sectional study

**DOI:** 10.1186/s40352-023-00240-x

**Published:** 2023-09-12

**Authors:** Ingie Osman, Antonio Williams, Katie Pierson, Eric Ryu, Rebecca J. Shlafer

**Affiliations:** 1 Department of Pediatrics, University of Minnesota, 717 Delaware St. SE, Minneapolis, MN 55414 USA; 2 COVID-19 Vaccine Confidence Advisory Board, University of Minnesota, Minneapolis, MN USA; 3 Humphrey School of Public Affairs, University of Minnesota, Minneapolis, MN USA

**Keywords:** COVID-19, Incarceration, Prison, Vaccination, Health promotion, Health equity

## Abstract

**Background:**

The COVID-19 pandemic has disproportionately impacted individuals in carceral facilities – both incarcerated people and staff. Vaccination is an important tool in reducing the risk of COVID-19 infection, hospitalization, and death. While the importance of promoting vaccination is clear, there are considerable barriers to doing so. This study aims to better understand: (1) why individuals chose to receive the COVID-19 vaccine; (2) why individuals were hesitant to vaccinate; (3) what motivators might influence a person’s decision to get vaccinated; and (4) what sources of information about COVID-19 vaccination people trust.

**Methods:**

We conducted a survey of incarcerated people and facility staff in three, large state prisons in Minnesota to identify barriers and facilitators to COVID-19 vaccination. Facilities were recruited to participate through purposive sampling, and surveys were administered between November and December 2021. Descriptive statistics were calculated using Stata.

**Results:**

Findings demonstrate that, for incarcerated individuals (*N* = 1,392), the most common reason for getting vaccinated was to return to normal activities in prison (61%, *n* = 801); the most common reason for being hesitant to get vaccinated was “other” (41%, *n* = 342), with individuals citing a variety of concerns. For staff (*N* = 190), the most common reason for getting vaccinated was to protect the health of family and friends (79%, *n* = 114); the most common reasons for being hesitant were disbelief that vaccination is necessary (55%, *n* = 23) and distrust of healthcare and public health systems (55%, *n* = 23). Incarcerated individuals reported that monetary and programmatic incentives would help motivate them to get vaccinated, while staff members said speaking with healthcare professionals and monetary incentives would help motivate them. Lastly, trusted sources of information for incarcerated individuals were healthcare professionals outside of prisons and jails, along with friends and family members. Staff members reported that they trusted healthcare professionals and national health organizations for information about COVID-19 vaccination.

**Conclusions:**

While considerable barriers to COVID-19 vaccination persist among both incarcerated individuals and staff members, these findings also highlight areas of intervention to increase COVID-19 vaccine confidence and promote health equity among those disproportionately impacted by the COVID-19 pandemic.

## Background

Since March 2020, there have been over 600,000 COVID-19 cases among people incarcerated in United States (US) correctional and detention facilities, and over 3,000 COVID-19-related deaths (Centers for Disease Control and Prevention, 2022a). The conditions of carceral facilities – overcrowding, poor ventilation, unsanitary environments – increase the risk of contracting and transmitting COVID-19 among those who are incarcerated, as well as staff (Dumont et al., 2012; Nowotny, Seide et al., 2021; Reinhart & Chen, 2021; Ward et al., 2021; Williams et al., 2020). Additionally, high rates of chronic health conditions and reduced capacity for health care services increase the risk of severe illness from COVID-19 infections (Binswanger et al., 2009; Maruschak et al., 2016). Throughout the pandemic, COVID-19 incidence and mortality rates in prisons and jails have been consistently higher than in the general population, with a 5.5 times higher risk of contracting COVID-19 and a 3 times higher risk of dying from COVID-19, adjusting for age, sex, and race/ethnicity (Saloner et al., 2020).

Because of systemic racism in the criminal legal system, people of color experience the impacts of incarceration more often and more profoundly (Bailey et al., 2017). People of color are incarcerated at disproportionately higher rates than white people in the US (Hartney & Vuong, 2009; Wendy, 2020). Nationally, the impacts of COVID-19 in carceral settings are exacerbated among people of color, who face a greater burden of COVID-19 infection and mortality, related to historical and contemporary racism and oppression (Adhikari et al., 2020; Centers for Disease Control and Prevention, 2022c; LeMasters et al., 2022; Muñoz-Price et al., 2020). Given this, addressing COVID-19 infection and transmission in carceral facilities is fundamentally an issue of health equity (Nowotny, Bailey et al., 2021).

One important tool in promoting health and wellbeing during the COVID-19 pandemic is vaccination. COVID-19 vaccines have been found to significantly reduce the risk of COVID-19 infection, hospitalization, and death (León et al., 2022; Scobie et al., 2021; Xu et al., 2021). In congregate environments with high rates of COVID-19 transmission, it is important to promote COVID-19 vaccine confidence and access for both incarcerated individuals and staff working in these settings. Staff working in prison and jail environments can carry COVID-19 into facilities from their communities and/or into their communities from facilities, posing a public health threat to both people who are incarcerated and others in the community (Wallace et al., 2021). Because we cannot predict staff’s adherence to COVID-19 guidelines in their personal lives, along with the varying levels of community transmission rates where staff members reside, there is considerable risk of staff bringing COVID-19 into facilities, increasing the risk of exposure to incarcerated people. This interconnectedness makes it critical to focus on promoting COVID-19 vaccine confidence among staff, in addition to people who are incarcerated.

While the importance of promoting vaccine confidence among these populations is clear, there are many challenges to doing so. Early reports found low vaccine uptake among staff in correctional facilities, attributed to concerns around short- and long-term side effects of vaccination, conspiracy theories, and distrust of the prison administration and its handling of the virus (Bertram & Sawyer, 2021; Lewis & Sisak, 2021).

In a sample of incarcerated populations in the Northwest, West, South, and Southeast regions of the country prior to the availability of COVID-19 vaccines, Stern and colleagues found that the most common reason for refusal of the COVID-19 vaccine was distrust of medical, correctional, or government institutions (Stern et al., 2021). Another study examining vaccine hesitancy among incarcerated individuals in Northern California jails found that incarcerated individuals indicated concerns about efficacy and side effects of the COVID-19 vaccine (Liu et al., 2022). In a study examining vaccination rates among people incarcerated in federal facilities, Hagan and colleagues found that factors associated with lower vaccination acceptance included younger age, female sex, non-Hispanic Black and Asian race/ethnicity, and having few underlying medical conditions (Hagan et al., 2021). Given the legacy of structural racism, historical and contemporary mistreatment of incarcerated individuals, and limited access to information while incarcerated, these findings emphasize the nuanced challenges to promoting COVID-19 vaccination among incarcerated populations. This study adds to a small, but growing body of research by examining barriers and facilitators to COVID-19 vaccination, by surveying both staff and incarcerated individuals from the same three Midwest state prisons, and surveying individuals after COVID-19 vaccines had been available for close to a year.

The objective of this study was to explore barriers and facilitators to COVID-19 vaccination among incarcerated people and staff. We use cross-sectional survey data with incarcerated people and staff in three Minnesota state prisons to better understand: (1) why individuals chose to receive the COVID-19 vaccine; (2) why individuals were hesitant to receive the COVID-19 vaccine; (3) what motivators might influence a person’s decision to get vaccinated; and (4) what sources of information about COVID-19 vaccination people trust.

## Methods

Our project team, in partnership with our community advisory board and administrators at the Minnesota Department of Corrections (MnDOC), conducted a survey of incarcerated people and facility staff in three state prisons to identify key barriers and facilitators to COVID-19 vaccination among these populations and guide the selection of our interventions. At the time the surveys were conducted, vaccines had been available for approximately 10 months, and booster doses were beginning to be rolled out.

### Study sites

To give us a broad range of experiences and responses, while balancing the burden of data collection on prisons during the pandemic, we purposively sampled incarcerated individuals and staff members from three Minnesota Correctional Facilities (MCF): the men’s intake facility (MCF-St. Cloud), women’s intake facility and the state’s only prison for women (MCF-Shakopee), and the facility with the lowest rates of vaccination at the time of data collection (MCF-Rush City). Approximate COVID-19 vaccination rates by facility are shown in Table 1.

### Data collection

In partnership with our community advisory board and administrators at the MnDOC, we designed and administered cross-sectional surveys with incarcerated adults and staff working in three state prisons. Prior to administering these surveys, we obtained approval from the MnDOC’s Research and Evaluation Advisory Council (REAC). The UMN Institutional Review Board (IRB) determined that the project constituted a surveillance project that provides timely situational awareness during a public health crisis and was therefore not research involving human subjects as defined by Department of Health and Human Services (DHHS) and Food and Drug Administration (FDA) regulations (U.S. Department of Health and Human Services, 2018).

#### Surveys with prison staff members

Wardens identified a staff member at each facility to assist with participant recruitment; this staff member then shared the web-based survey (using Qualtrics) via email to all staff members at each facility. We did not make a distinction in the staff roles that were recruited; thus, any individual the DOC identified as staff and shared the survey with was eligible to complete the survey. The survey was open between November 17, 2021 and December 9, 2021. Staff members who participated could opt into a drawing for a chance to win one $100 gift card per facility. Surveys were anonymous, but staff members who participated had the option to fill out a separate form with their contact information to be eligible in the gift card drawing.

#### Surveys with incarcerated individuals

Wardens identified a staff member at each facility to assist with participant recruitment and survey administration. Staff administered the survey at each facility on one single day between November 23, 2021 and December 2, 2021. Ahead of the survey administration date, incarcerated individuals were notified about the survey opportunity through the facility’s Memo of the Day. Using a detailed survey protocol, the designated staff member shared one paper survey with each incarcerated individual, and placed a drop-off box for completed surveys in each living unit. Surveys were made available in English, Spanish, and Mandarin; translations were completed by a professional translation company that utilizes quality checks and validation processes to ensure accuracy.

Every individual who completed the survey had the option to receive a $5 account credit for their participation; if participants wanted to be compensated for their participation, they were asked to put their unique identification number on the bottom of their survey. All completed surveys were mailed back to the researchers at the University of Minnesota. After confirming there were no duplicate identification numbers and documenting which accounts to add the $5 credit to, all identification numbers were removed from the surveys for subsequent processing, data entry, and analysis. A team of University of Minnesota undergraduate and graduate student research assistants, trained in research confidentiality and data storage, entered responses into Qualtrics. To minimize data entry errors, student research assistants directly entered data into a Qualtrics form that had structured response fields. Then, project staff ran descriptive statistics for the entire dataset to identify any data discrepancies or implausible responses.

### Measures

#### Surveys with prison staff members

The survey combined both closed-ended and open-ended questions about COVID-19 vaccination, modeled on the CDC COVID-19 Vaccine Confidence: Rapid Community Assessment Tool, and was tailored to populations working in prison settings (Centers for Disease Control and Prevention, 2022b). This survey consisted of 20 questions posed to staff members on their demographic characteristics, COVID-19 vaccination status, facilitators and barriers to COVID-19 vaccination, motivators to get vaccinated, trusted sources of information, and employer COVID-19 vaccination policies. When asked about facilitators and barriers, motivators, and trusted sources of information, participants were able to select more than one option. A series of seven Likert scale questions were included focused on confidence in COVID-19 vaccination, social support around vaccination, information about COVID-19 vaccines, and making health decisions around COVID-19 vaccination. Two open-ended questions asked about staff members’ questions related to COVID-19 vaccination and ideas for interventions to improve COVID-19 vaccine confidence among prison staff.

#### Surveys with incarcerated individuals

Similar to the survey for staff members, this survey combined both closed-ended and open-ended questions about COVID-19 vaccination, modeled on the CDC COVID-19 Vaccine Confidence: Rapid Community Assessment Tool, and was tailored to incarcerated populations (Centers for Disease Control and Prevention, 2022b). The project’s community advisory board, composed of 14 formerly incarcerated individuals, worked to tailor survey questions in partnership with project staff and helped pre-test materials. This survey consisted of 10 questions posed to incarcerated individuals on their demographic characteristics, COVID-19 vaccination status, facilitators and barriers to COVID-19 vaccination, motivators to get vaccinated, and trusted sources of information. When asked about facilitators and barriers, motivators, and trusted sources of information, participants were able to select more than one option. A series of five Likert scale questions were included focused on confidence in COVID-19 vaccination, social support around vaccination, information about COVID-19 vaccines, and making health decisions around COVID-19 vaccination. Three open-ended questions asked about incarcerated individuals’ questions related to COVID-19 vaccination, ideas for interventions to increase COVID-19 vaccine confidence for incarcerated people, and any other comments and concerns.

### Analyses

Quantitative survey data were exported from Qualtrics to Stata for analysis. Descriptive statistics, including frequencies and percentages, were calculated for demographic characteristics and COVID-19 vaccination-specific variables. Statistical analyses were performed using Stata version 15 (StataCorp, 2017). Missing data across all study variables was limited, ranging from 0.2 to 10% on categorical variables. Descriptive statistics were reported out of the total data available for each question.

All open-ended responses were reviewed and grouped into salient themes regarding common questions and ideas to increase COVID-19 vaccine confidence. These salient themes were shared with the project’s community advisory board for feedback and assistance with interpretation.

## Results

### Surveys with prison staff members

All staff members at MCF-Shakopee, MCF-St. Cloud, and MCF-Rush City were invited to take this brief, web-based survey about COVID-19 vaccination. In order to participate in this survey, individuals had to be employed by the Minnesota Department of Corrections (MnDOC). The final sample size for analysis was 190 participants. Of the three facilities that participated, MCF-Shakopee had the highest response rate (24%) (Table 1).

Table 2 reports the demographic characteristics for our sample of staff members. The majority of participants identified as women (61%) and non-Hispanic white (89%). The average age of survey participants was 44.3 years (*SD* = 9.5). 76% of respondents reported having received one or more doses of a primary series of the COVID-19 vaccine, and 37% of respondents reported having received a COVID-19 vaccine booster dose.

**Table 1:** Facility characteristics

	MCF-Shakopee	MCF-St. Cloud	MCF-Rush City
Security level	All security levels	Level 4 Close**	Level 4 Close**
Incarcerated population size*	403	723	893
Staff population size*	262	421	315
Survey response rate			
Incarcerated population	360 (89%)	579 (80%)	453 (51%)
Staff members***	64 (24%)	68 (16%)	43 (14%)
Approximate COVID-19 vaccination rate at the time of the survey****			
Incarcerated population	77%	70%	63%
Staff members	74%	57%	45%

*Population size was calculated at the time data collection occurred at each facility, in late November and early December of 2021

**Level 4 close security level refers to facilities that are one level below maximum security

***Our dataset is missing the facility for 15 staff members; we report all available data

****Approximate COVID-19 vaccination rate (full series) was shared by the Minnesota Department of Corrections in September 2021

**Table 2:** Demographic characteristics of sample and COVID-19 vaccination status

	Staff working in MN prisons *n* (%)	People incarcerated in MN prisons *n* (%)
Gender identity		
Man	66 (37%)	1,007 (72%)
Woman	109 (61%)	350 (25%)
Self-described	4 (2%)	23 (2%)
Transgender	0 (0%)	10 (1%)
Non-binary	0 (0%)	7 (< 1%)
Race/ethnicity		
Non-Hispanic white	160 (89%)	562 (41%)
Non-Hispanic Black or African American	1 (< 1%)	267 (19%)
Multi-racial	8 (4%)	209 (15%)
American Indian or Alaska Native	1 (< 1%)	139 (10%)
Hispanic, Latino, Latina, Latine	0 (0%)	83 (6%)
Self-described	0 (0%)	66 (5%)
Asian or Asian American	2 (1%)	38 (3%)
Native Hawaiian or Other Pacific Islander	0 (0%)	5 (< 1%)
Middle Eastern or North African	0 (0%)	3 (< 1%)
Prefer not to answer	9 (5%)	1 (< 1%)
Mean age	44.3 years (*SD* = 9.5)	37.1 years (*SD* = 10.7)
COVID-19 vaccination status		
Received one or more doses of a primary series of the COVID-19 vaccine	143 (76%)	1,178 (85%)
Received a booster dose of the COVID-19 vaccine	69 (37%)	N/A

**Fig. 1:**
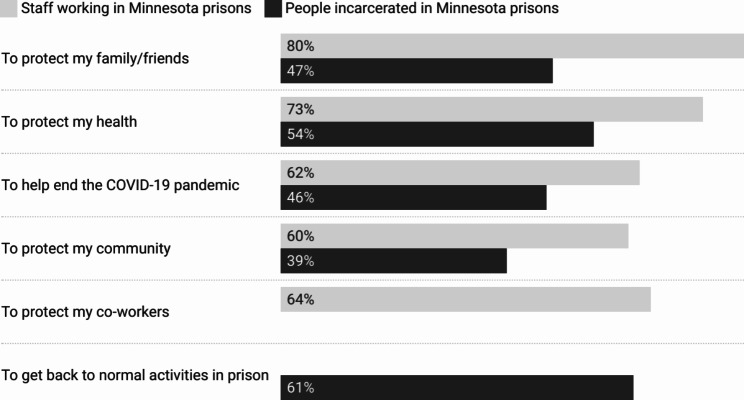
Top 5 reasons for getting vaccinated among staff and people incarcerated in Minnesota prisons

**Fig. 2:**
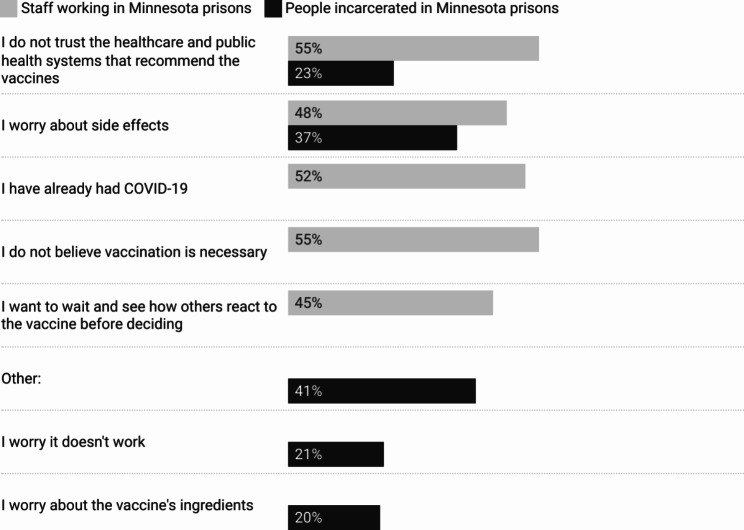
Top 5 reasons for not getting or being unsure about getting vaccinated among staff and people incarcerated in Minnesota prisons

Of those who had gotten or considered getting the COVID-19 vaccine, the most commonly cited reason was to protect the health of family and friends (79%, *n* = 114). Many also wanted to protect their own health (73%, *n* = 105), the health of coworkers (64%, *n* = 92), and wanted to help end the COVID-19 pandemic (62%, *n* = 89) (Fig. 1). The two most common reasons cited for refusal or hesitation were disbelief that the vaccination is necessary (55%, *n* = 23) and distrust of the healthcare and public health systems that are recommending the vaccines (55%, *n* = 23). A little over half of respondents also stated they do not want to or are unsure about getting vaccinated because they already had COVID-19 (52%, *n* = 22). Other respondents cited concerns about side effects (48%, *n* = 20) and concerns about the vaccine’s ingredients (45%, *n* = 19) (Fig. 2).

When asked about motivations to get vaccinated, 39% (*n* = 44) of respondents said that speaking with healthcare and public health professionals about the vaccine would help motivate them, 35% (*n* = 40) said monetary incentives would help motivate them, and 26% (*n* = 30) said reading educational materials about COVID-19 vaccines would help motivate them to get vaccinated. Most (87%, *n* = 123) respondents said healthcare professionals were a trusted source of information about COVID-19 vaccination. A little less than half (44%, *n* = 63) said national health organizations would be another trusted source of information, and 39% (*n* = 55) said they would trust local or state health departments for information about COVID-19 vaccination.

### Surveys with incarcerated individuals

All incarcerated individuals at MCF-Shakopee, MCF-St. Cloud, and MCF-Rush City were invited to take this brief, paper-based survey about COVID-19 vaccination. In order to participate in this survey, individuals had to be currently incarcerated in one of the three MnDOC facilities, and be able to read/write in English, Spanish, or Mandarin. The final sample size for analysis was 1,392 participants. Of the three facilities that participated, MCF-Shakopee had the highest response rate (89%) (Table 1).

Table 2 presents demographic characteristics for the sample of incarcerated individuals. The majority of participants identified as men (72%) and the average age of participants was 37 years (*SD* = 10.7). 41% of participants identified as non-Hispanic white, 19% identified as non-Hispanic Black, 15% identified as multiracial, 10% identified as American Indian or Alaska Native, and the remainder of participants identified as Hispanic, another race/ethnicity, Asian or Asian American, Native Hawaiian or other Pacific Islander, or Middle Eastern or North African. Most (85%) participants reported having received one or more doses of the COVID-19 vaccine.

Of those who had gotten or considered getting the vaccine, the most commonly cited reasons were to get back to normal activities in prison (61%, *n* = 801) and to protect their own health (54%, *n* = 704). Many also wanted to protect their family and friends (47%, *n* = 618), help end the COVID-19 pandemic (46%, *n* = 605), and protect their community (39%, *n* = 510) (Fig. 1). Of those who had not gotten or were hesitant about getting the vaccine, the most cited reason (41%, *n* = 342) was “Other,” where participants could write in additional responses. These responses included concerns about breakthrough cases, distrust in government, emphasizing one’s own personal choice about vaccination, and sharing concerns about vaccine development. Additional reasons for not getting or being hesitant about getting the COVID-19 vaccine included concerns over vaccine side effects (37%, *n* = 304), distrust of the healthcare and public health systems that recommend the vaccines (23%, *n* = 187), concerns that the vaccines do not work (21%, *n* = 177), and concerns about the vaccine’s ingredients (20%, *n* = 168) (Fig. 2).

When asked what specific incentives would motivate them to get vaccinated, the most common responses were receiving money (53%, *n* = 665), being able to participate in prison programming (52%, *n* = 655), and receiving commissary credit or credits for phone calls and emails (36%, *n* = 452). When asked who they would trust as a source of information about the COVID-19 vaccination, most cited healthcare professionals *outside of* prisons and jails (54%, *n* = 681), friends and family (50%, *n* = 632), and national health organizations (40%, *n* = 502).

## Discussion

Through this cross-sectional survey with incarcerated people and staff in three Minnesota state prisons, we worked to explore barriers and facilitators to COVID-19 vaccination and better understand: (1) why individuals chose to receive the COVID-19 vaccine; (2) why individuals were hesitant to receive the COVID-19 vaccine; (3) what motivators might influence a person’s decision to get vaccinated; and (4) what sources of information about COVID-19 vaccination people trust.

These findings demonstrate that although this sample of staff members and incarcerated individuals in Minnesota prisons had relatively high COVID-19 vaccination rates, considerable barriers to COVID-19 vaccine confidence remain among these populations. Although some COVID-19 vaccine promotion activities occurred across all facilities, including DOC vaccine clinics, a video from the DOC’s medical director, and free coffee as a vaccine incentive, survey responses reflected that individuals’ questions persisted around whether the vaccine was necessary, whether prior infection offered protection, the severity of side effects, the safety of vaccine ingredients, and whether the vaccine was effective. For incarcerated people in particular, the limited flow of information into facilities and lack of autonomy in selecting information sources makes it especially challenging to make informed decisions about health and wellbeing, and may contribute to these lingering questions and concerns. Sharing timely and transparent information about COVID-19 vaccination is essential as the COVID-19 pandemic continues to evolve.

A major cause for concern for both groups was mistrust in the public health and healthcare agencies that recommend COVID-19 vaccination. Mistrust, particularly among the incarcerated population, relates to centuries of oppression, medical racism, violence, and experimentation on behalf of medical and carceral institutions (Benkert et al., 2006; Elumn et al., 2021; Scharff et al., 2010; Vandergrift & Christopher, 2021). Acknowledging the rightful hesitation by groups that have been harmed by these systems is essential to engaging in efforts to promote COVID-19 vaccine confidence.

Findings also indicate that tapping into trusted sources to share information about COVID-19 vaccination is important for both populations. Among incarcerated individuals, healthcare professionals *outside of* prisons and jails, along with friends and family, were most commonly cited as trusted sources of information. This reflects the common theme of rightful mistrust that this population has, given what we know about provision of health care provided in carceral facilities, centuries of harm that has occurred, along with little autonomy in healthcare decision-making that people experience behind bars (Brinkley-Rubinstein, 2013; Elumn et al., 2021; Jolin et al., 2022; Plugge et al., 2008; Vandergrift & Christopher, 2021; Wilper et al., 2009). Engaging trusted sources of health information outside of carceral facilities, including family members, community members, and healthcare providers is needed through and beyond the COVID-19 pandemic and should be met with necessary investments in communication, programming, and telehealth.

Over half of staff members who had not gotten or were unsure about getting the COVID-19 vaccine expressed distrust in public health and healthcare systems recommending vaccination. This is staggering when we consider the implications that this has on the health and wellbeing of not just staff, but the incarcerated individuals who interact with staff, as well. Yet, when staff members were asked who they trusted as sources of information about COVID-19 vaccines, most said healthcare professionals, followed by national health organizations and local or state health departments. Future work is needed to disentangle these seemingly contradictory responses, and better understand the nuances related to what sources of information are most trusted by prison staff members and how to best communicate evidence-based, public health messaging to this population.

Finally, survey responses indicated that different forms of incentives might serve as facilitators to COVID-19 vaccination. The majority of incarcerated individuals reported that both monetary and programmatic incentives would motivate them to get vaccinated. Some staff members reported that speaking with healthcare providers about vaccination, as well as monetary incentives, would help motivate them to get vaccinated.

### Recommendations

These data provide key insights for Department of Corrections (DOC) working to promote vaccination efforts among staff and incarcerated populations. Below are a series of recommendations that work towards increasing COVID-19 vaccine confidence.

1. *Develop channels to share timely and evidence-based information about COVID-19 vaccination and other rapidly-evolving public health emergencies.* Other states (e.g., Massachusetts, Pennsylvania) have effectively accomplished this in a number of ways, including sharing timely updates about the COVID-19 pandemic and vaccination efforts by hosting regular “town-hall” meetings with staff, incarcerated populations, and loved ones, and hosting regular Q&A sessions with trusted medical providers and outside experts to answer questions and share timely information as the COVID-19 pandemic continues to evolve (Erfani et al., 2022; Pennsylvania Department of Corrections, 2022). We recommend all states develop multiple communication channels to communicate timely, evidence-based information to diverse audiences impacted by incarceration during public health emergencies.
  Other successful efforts have included developing tailored educational materials, both in video and print format, to address common questions and concerns about COVID-19 vaccination from staff and incarcerated populations (DaSilva & Fish, 2021). Additional efforts to promote timely communication could include offering free phone calls, video visits, and/or emails for all incarcerated individuals to stay connected to those in the community who can share up-to-date information (Bertram, 2020). A combination of these approaches is recommended to reach as many individuals with information as possible.
2. *Tap into trusted sources to share information about COVID-19 vaccination in addition to modeling evidence-based practices.* Given the rightful mistrust that many incarcerated people feel towards healthcare and carceral systems, it is incredibly important to lean on trusted sources to deliver timely and reliable information. Peer education and peer support programs in prison settings have been associated with reductions in risky behaviors and other positive health effects (Bagnall et al., 2015). Developing or adapting models of peer support to share information about COVID-19 vaccination has the potential to build trust and increase confidence in COVID-19 vaccination (Kraft, 2021). Individuals serving in a peer educator role should be compensated for their efforts, should receive professional support for their defined role, and should have modes of accessing timely, evidence-based information as the COVID-19 pandemic (and other public health emergencies) evolve.
  Other states have utilized family members, loved ones, outside experts, and volunteers as trusted sources of information for both staff and incarcerated individuals (Baumgarten, 2021; Kraft, 2021). Leaning into trusted sources of information outside of prisons should be paired with investments in making communication, video programming, telehealth, and other forms of engagement free for incarcerated individuals and their loved ones.
  In addition to engaging trusted sources of information, it is also imperative that healthcare professionals inside DOCs model, implement, and adhere to COVID-19 prevention, mitigation, and treatment standards of care and evidence-based practices. Modeling these practices is important in building trust around health information within DOCs.
3. *Promote COVID-19 vaccination and other preventive health measures by utilizing incentives.* Financial incentives have been shown to increase adherence to vaccination among the general population (Higgins et al., 2021). Paired with investments in education and efforts to build trust, incentives have helped other states reach high levels of COVID-19 vaccine coverage among incarcerated individuals and staff members (“Delaware Inmates Offered Incentives to Get COVID Vaccine,” 2021; “Public Safety Credits Incentives for Rise in Inmate Vaccinations,” 2021; Holloway, 2021; Kruegel, 2021). Given the ongoing restrictions within prisons, increasing access to programming as an incentive was viewed favorably among incarcerated individuals, as well. We recommend that if DOCs do implement incentives, those incentives must center on the principles of equity and autonomy, and be non-coercive or exploitative. In order to achieve this, there is a need for an ethical evaluation of incentives in carceral settings; we recommend the development of an advisory network to further examine best practices around the ethical use of incentives for health promotion among incarcerated populations.
4. *Acknowledge the rightful mistrust towards public health, healthcare, and carceral institutions.* Our survey responses highlight that incarcerated individuals have considerable mistrust in public health, healthcare, and carceral systems and feel as though these systems are not trustworthy sources of information. Research has shown that health communications must be tailored to acknowledge the impact that social injustice, health inequities, and systemic racism has on mistrust (Dong et al., 2022; Marcelin et al., 2021). As public health professionals and DOCs develop strategies to increase COVID-19 vaccination, or behavior changes around any health issue, acknowledging mistrust is essential to the process of changing perspectives.
  While taking steps to acknowledge this ongoing mistrust and trauma at the hands of systems is important, it is also important to note that as long as incarceration and other systems that cause harm persist, so too, will this mistrust. Mistrust cannot solely be addressed on an individual level, and must continue to be addressed at institutional, governmental, and policy levels. We must shift our understanding of public health and its intersections with carceral involvement and systemic racism to better understand how these systems can undermine community health and wellbeing.

### Limitations

Multiple limitations to this study are worth noting. First, potential biases, such as socially desirable response bias, may have impacted participant responses. Second, our sample was not entirely representative of all adults working or incarcerated in Minnesota prisons, given our purposive sampling of three facilities. Staff respondents were disproportionately white, women, and slightly older compared to the demographic makeup of all staff in each of the three facilities. Our sample of incarcerated individuals contained more women, fewer white individuals, and was slightly younger compared to incarcerated people across all MnDOC facilities. Across both surveys, a key limitation that warrants further inquiry is examining these barriers and facilitators by key demographic characteristics, including race and ethnicity, gender identity, facility type, and staff roles. Lastly, a notable limitation of this study was the much lower response rate among staff relative to incarcerated people in each of the three prisons; staff survey results are therefore limited in generalizability. While the reasons for this low response rate are unknown, the COVID-19 pandemic has put a strain on staff; it’s possible that staff members were fatigued and therefore disengaged in this type of research opportunity. A deeper understanding of the pandemic’s broad impacts on staff health and wellbeing, and occupational health hazards, is an area for further inquiry. Other future work examining how perceptions related to different vaccine types (i.e., mRNA, viral vector, and protein subunit vaccines) may vary among staff and incarcerated populations should be explored, as well.

## Conclusions

Results from this survey highlight key barriers and facilitators to COVID-19 vaccination, along with areas of intervention that have the potential to increase COVID-19 vaccine confidence and uptake among staff working in prisons and people who are incarcerated. Understanding barriers and facilitators to COVID-19 vaccination among these populations is critical to developing interventions to address pandemic-related health disparities and promote health equity among those disproportionately impacted by the COVID-19 pandemic.

## Data Availability

The dataset analyzed in the current study is available from the corresponding author on reasonable request.

## Abbreviations

CDC: Centers for Disease Control and Prevention
COVID-19: Coronavirus disease 2019
DHHS: Department of Health and Human Services
DOC: Department of Corrections
FDA: Food and Drug Administration
IRB: Institutional Review Board
MCF: Minnesota Correctional Facility
MN: Minnesota
MnDOC: Minnesota Department of Corrections
REAC: Research & Evaluation Advisory Council
US: United States
